# Poor Corticospinal Motor Neuron Health Is Associated with Increased Symptom Severity in the Acute Phase Following Repetitive Mild TBI and Predicts Early ALS Onset in Genetically Predisposed Rodents

**DOI:** 10.3390/brainsci11020160

**Published:** 2021-01-26

**Authors:** Mor R. Alkaslasi, Noell E. Cho, Navpreet K. Dhillon, Oksana Shelest, Patricia S. Haro-Lopez, Nikhil T. Linaval, Josh Ghoulian, Audrey R. Yang, Jean-Philippe Vit, Pablo Avalos, Eric J. Ley, Gretchen M. Thomsen

**Affiliations:** 1 Board of Governors Regenerative Medicine Institute, Cedars-Sinai Medical Center, Los Angeles, CA 90048, USA; mor.alkaslasi@cshs.org (M.R.A.); noell.cho@cshs.org (N.E.C.); roksana.elder@cshs.org (O.S.); patricia.harolopez@cshs.org (P.S.H.-L.); pablo.avalos@cshs.org (P.A.); 2 Department of Surgery, Division of Trauma and Critical Care, Cedars-Sinai Medical Center, Los Angeles, CA 90048, USA; navpreet.dhillon@cshs.org (N.K.D.); nikhil.linaval@cshs.org (N.T.L.); josh.ghoulian@cshs.org (J.G.); audrey.yang@cshs.org (A.R.Y.); eric.ley@cshs.org (E.J.L.); 3 Biobehavioral Research Core, Cedars-Sinai Medical Center, Los Angeles, CA 90048, USA; Jean-Philippe.Vit@cshs.org

**Keywords:** amyotrophic lateral sclerosis, traumatic brain injury, concussion, corticospinal motor neurons, chronic traumatic encephalopathy

## Abstract

Traumatic brain injury (TBI) is a well-established risk factor for several neurodegenerative disorders including Alzheimer’s disease and Parkinson’s disease, however, a link between TBI and amyotrophic lateral sclerosis (ALS) has not been clearly elucidated. Using the SOD1^G93A^ rat model known to recapitulate the human ALS condition, we found that exposure to mild, repetitive TBI lead ALS rats to experience earlier disease onset and shortened survival relative to their sham counterparts. Importantly, increased severity of early injury symptoms prior to the onset of ALS disease symptoms was linked to poor health of corticospinal motor neurons and predicted worsened outcome later in life. Whereas ALS rats with only mild behavioral injury deficits exhibited no observable changes in corticospinal motor neuron health and did not present with early onset or shortened survival, those with more severe injury-related deficits exhibited alterations in corticospinal motor neuron health and presented with significantly earlier onset and shortened lifespan. While these studies do not imply that TBI causes ALS, we provide experimental evidence that head injury is a risk factor for earlier disease onset in a genetically predisposed ALS population and is associated with poor health of corticospinal motor neurons.

## 1. Introduction

Amyotrophic lateral sclerosis (ALS) is a neurodegenerative disease characterized by a progressive loss of motor neurons leading to paralysis and death within 3 to 5 years of disease onset. No effective treatments exist, and its exact etiology is still under investigation. Mutations in over 30 genes have been identified that lead to the familial form of ALS, including the Cu/Zn *superoxide dismutase-1 (SOD1)* gene [1], which accounts for about 20% of the familial cases of ALS. Still, the majority of ALS cases are sporadic, with no known origin.

While degeneration in both upper and lower motor neurons occurs in ALS patients and animal models, the exact origin of disease is unknown. Recent studies have found an early role for upper motor neurons in the brain in the initiation of disease [2,3,4,5]. However, despite convincing evidence of brain involvement in initiating motor circuitry breakdown, there is much to be understood regarding the role of the brain in ALS pathogenesis.

Much of the etiology of ALS is likely rooted in complex interactions between genetic and environmental risk factors. Various risk factors that may be associated with ALS have been explored, including previous exposure to heavy metals and organic chemicals, and a history of head trauma [6]. Many studies have implicated a history of Traumatic brain injury (TBI) as a significant risk factor in the development of neurodegenerative disorders such as Alzheimer’s disease (AD) and other dementias [7,8,9,10], Parkinson’s disease (PD) [11,12], and ALS [6,13,14]. Interestingly, the assessment of neurodegenerative disease occurrence among contact sports athletes such as American football players shows a three-fold increase in mortality caused by neurodegeneration relative to the general US population [15]. Recurrent TBI (rTBI) is causatively linked to Chronic Traumatic Encephalopathy (CTE) [16,17], a neurodegenerative disease characterized by brain atrophy and upregulated cortical tau, supporting the capacity for TBI to instigate neurodegeneration. This evidence, coupled with the support for early brain involvement in disease onset, emphasizes the necessity for further investigation of the temporal and pathological effects of repetitive TBI on the onset of ALS.

As the awareness of the association between head trauma and ALS grows, and as larger data sets are evaluated, the potential to understand this link in much greater detail appears tenable. Here, we identified a clear association between head injury and early onset of ALS in genetically predisposed rodents and provide the degeneration of corticospinal motor neurons as an underlying cause.

## 2. Materials and Methods

### 2.1. Animals

Wildtype (WT) and SOD1^G93A^ (“SOD1”, herein) transgenic rats of Sprague-Dawley background were housed under National Institutes of Health guidelines and all experiments were conducted in accordance with the Cedars-Sinai Medical Center Institutional Animal Care and Use Committee (IACUC) guidelines under IACUC protocol #006771. The SOD1 rat used in these studies is commonly used to model ALS in that it exhibits paralysis in the forelimbs and/or hindlimbs that recapitulates human ALS pathology^1^.

### 2.2. Experimental Groups

Forty WT and 65 SOD1 rats were included in these studies. A mild, bilateral, closed-skull, controlled cortical impact (CCI) injury was administered once per week for 5 weeks beginning at postnatal day (P) 60 in 23 WT and 36 SOD1 rats. Sham injury was performed on 17 WT and 29 SOD1 rats. SOD1 rats were euthanized when they reached disease endpoint, defined as when they could no longer right themselves within 30 s of being placed on their side. A subset of WT rats was pre-selected for euthanasia at 48 to 72 h after final injury (“acute”, age ~90 days) or 25 weeks after the first injury (“long”, age ~235 days). All TBI animals were further divided into subgroups of injury severity based on the degree of rotarod deficits assessed 6 weeks after the first injury (or 24 h after the fifth injury for the “acute” groups). Injury severities were classified as either “mild” (latency to fall: ≥ 50 s, total *n* = 32; WT, *n* = 14; SOD1, *n* = 18) or “severe” (latency to fall: <50 s, total *n* = 27; WT, *n* = 9; SOD1, *n* = 18). Both male and female rats were studied. When analyzed separately, there were no significant differences between male and female groups and data was therefore pooled.

### 2.3. Injury

A mild, bilateral, closed-skull, traumatic brain injury was administered at postnatal day 60 using a controlled cortical impact (CCI) device. Rats receiving injury were anesthetized with 2.5% isoflurane and positioned in a nose cone on a cloth pad. No stereotaxic restraint was used, however the nose cone acted to stabilize the head slightly. The cortical injuries were centered at bregma, 3 mm lateral to the midline, and delivered using a 5 mm diameter metal tip mounted on the CCI device at an angle of 10 degrees from the vertical plane. The impactor was driven at a velocity of 6 m/s at a depth of 4 mm and a dwell time of 0.2 ms. The first impact occurred on the left side followed by a second identical impact on the right side within 15 s of the first. Rats in the sham groups were anesthetized for the same amount of time as those in the TBI groups but were not administered injury. Injuries were delivered once a week for 5 weeks to allow for some recovery from the acute symptoms of mild TBI between injury events.

### 2.4. Rotarod

As a test of balance and motility, rats were placed on a rotating rod (3 inches in diameter, www.sandiegoinstruments.com, San Diego, CA, USA) for 210 s per trial. The speed of the rotation started at 3 revolutions/min, was constant for the first 30 s, and then accelerated progressively for 3 min to reach the speed of 30 revolutions/min. On each session day, the rats underwent three trials separated by about 30 min and the latency to fall between the three trials was averaged for analysis. Baseline testing was performed 1 week prior to the first injury, and post-injury testing occurred on day 6 after each injury session and continued weekly thereafter until end-point or time-point.

### 2.5. The Basso, Beattie, and Bresnahan Locomotor Rating Scale

The Basso, Beattie, and Bresnahan (BBB) scoring system [18] was used to assess an animal’s ability to walk around its environment and quantify the degree of paralysis of each limb. This 21-point scoring system is an open-field locomotor test of limb function—a score of 21 indicates coordinated limb movement, consistent toe clearance, and parallel paw placement, while a score of zero indicates no observable limb movement. A BBB score of 17 and above is normal for a healthy, nonparalyzed WT rat, while BBB scores below 15 indicate limb paralysis. BBB scores provide an indication of the timing of paralysis onset and degree of paralysis in each limb. These scores were assessed once weekly by an observer blinded for genotype and treatment starting the week before cortical injury until the animal’s endpoint.

### 2.6. Tissue Collection

Animals were euthanized by ketamine/xylazine cocktail administration, followed by transcardial perfusion with 0.9% saline. Brain tissue was collected and post-fixed in 4% paraformaldehyde overnight and stored in 30% sucrose. Fixed brains were sectioned coronally at 30 μm using a microtome and collected as free-floating sections for histology.

### 2.7. Immunohistochemistry

For immunofluorescent identification of corticospinal motor neurons, sections were stained for COUP-TF-interacting protein 2 (CTIP2). After blocking in 5% normal donkey serum and 0.25% Triton X-100 in phosphate-buffered saline (PBS) for 1 h at room temperature (RT), sections were incubated for two nights at 4 °C with rabbit polyclonal antibody to CTIP2 (1:500; Abcam, Cambridge, MA, USA). After washing, sections were incubated in an appropriate fluorescent secondary antibody at a concentration of 1:500 for 1 h at RT. CTIP2 stained sections were washed and further stained with Neurotrace (1:25; Life Technologies, Carlsbad, CA), a fluorescent Nissl stain, for 2 h at RT.

### 2.8. Stereological Analysis

Unbiased stereological analysis of CTIP2+ corticospinal motor neurons was performed on three 30 µm sections 720 µm apart starting at 1 mm posterior to bregma. Rectangular contours with a perimeter of 3400 µm were drawn on each section in the right hemisphere, 1500 µm from the midline, encompassing layer V of the motor cortex. The optical fractionator method was used with a counting frame of 100 μm × 100 μm and a grid size of 200 μm × 200 μm (yielding 13–20 counting sites per section). The nucleator method was used to measure cell size based on Neurotrace staining of the neuron cell body (MBF Bioscience software 2017, Williston, VT, USA).

### 2.9. Statistical Analysis

Statistical analyses were performed using GraphPad Prism software (version 7.0, San Diego, CA, USA). Student’s *t* tests, and one-way and two-way ANOVA using Bonferroni’s post hoc analyses were performed to determine standard error of the mean (SEM) with a 95% confidence level. Kaplan-Meier survival analyses were analyzed by the log-rank test, and comparisons of median disease durations and survival times were analyzed by the Wilcoxon’s signed-rank test.

## 3. Results

### 3.1. Repetitive Bilateral Mild TBI Results in Long Term Functional Deficits in WT and SOD1 Rats

**Figure 1:**
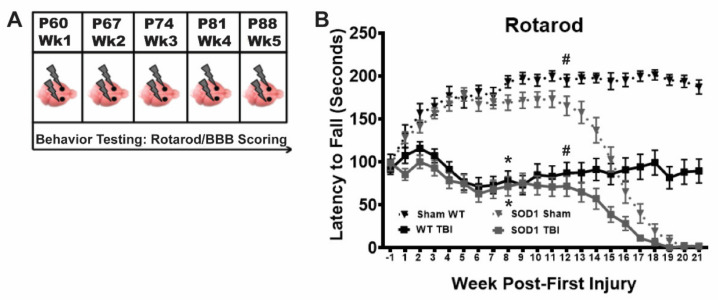
Repetitive bilateral mild traumatic brain injury (TBI) results in long term functional deficits in wildtype (WT) and SOD1 rats. (**A**) Rats in the TBI group received closed-skull bilateral controlled cortical impact (CCI) injuries to the motor cortex once a week for five weeks starting at postnatal day (P) 60 and behavior was monitored using Rotarod and Basso-Beattie-Bresnahan (BBB) scoring. (**B**) Both WT and SOD1 rats experiencing recurrent TBI (solid black and gray squares, respectively) exhibited significant early rotarod deficits, relative to both WT (dotted black triangle) and SOD1 (dotted gray triangles) sham. While TBI rats, regardless of genotype, appeared to recover following the second injury, after the third injury rotarod scores continued to decline and remained significantly lower than sham for the duration of the study. SOD1 rats in both sham and TBI exhibited disease-related progressive decline starting around week 13. (* SOD1 Sham vs SOD1 TBI, * WT sham vs WT TBI, # SOD1 Sham vs WT Sham, # SOD1 TBI vs WT TBI, Two-way ANOVA *p* < *0*.05).

We previously established a model of repetitive mild TBI in WT rats that results in sustained motor function deficits and CTE-like brain pathology [19,20]. Here, we tested this injury model in the SOD1^G93A^ ALS rat to determine if this type of injury leads to an earlier ALS disease phenotype. WT and SOD1 rats were administered closed-skull, bilateral, CCI injury once a week for 5 weeks and tested weekly on the rotarod for balance and motor function (Figure 1A). In addition, animals were monitored weekly for signs of ALS-like symptoms including motor deficits and paralysis by means of BBB scoring. TBI animals in both WT and SOD1 groups exhibited mild rotarod deficits after the first injury, relative to sham controls. All TBI animals recovered slightly after the second injury, but rotarod deficits continued to decline following the third injury and were never fully recovered in either the WT (tested out to 54 weeks, data not shown) or SOD1 TBI groups (Figure 1B). This indicates that it is likely that a minimum of two to three mild injuries is necessary to maintain this extent of permanent deficits. WT and SOD1 animals in the TBI group exhibited similar deficits on the rotarod over time, with a significant decline occurring after week 12 in both sham and TBI SOD1 rats that was associated with ALS disease onset.

### 3.2. Repetitive Bilateral Mild TBI Results in Long-Term Mild Paralysis in WT Rats and Earlier Disease Onset in SOD1 Rats

**Figure 2:**
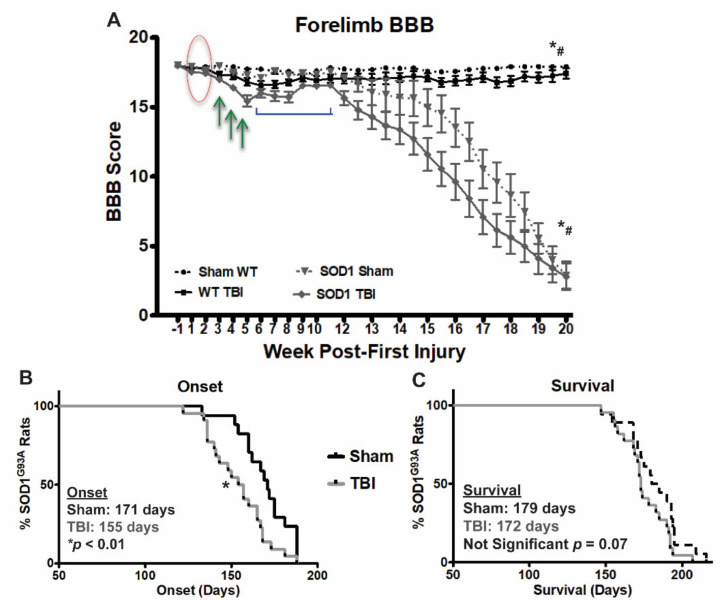
Repetitive bilateral mild TBI results in long-term mild paralysis in WT rats and earlier disease onset in SOD1 rats. (**A**) BBB scoring following repetitive mild TBI revealed no signs of paralysis after the first two weeks of injury (red oval). However, paralysis was observed following the 3rd injury and continued to worsen throughout the course of injury (green arrows). WT rats maintained a mild level of paralysis throughout the length of the study. An improvement in BBB score occurred following the final injury, prior to onset of disease in SOD1 rats (blue bracket). (* SOD1 sham SOD1 TBI, * WT sham vs WT TBI, # SOD1 Sham vs WT Sham, # SOD1 TBI vs WT TBI, Two-way ANOVA *p* < *0*.05) (**B**) Onset of paralysis based on forelimb BBB assessment occurred significantly earlier in SOD1 TBI rats, relative to SOD1 sham rats (155 vs. 171 days, respectively * *p* < 0.01). (**C**) Earlier onset of disease did not translate to significantly shortened survival time in SOD1 TBI rats when grouped as a whole, relative to SOD1 sham rats.

As rotarod is not a sensitive measure of ALS disease progression (as animals become sick, their performance declines sharply), BBB scoring was used to assess paralysis and more precise progressive forelimb decline. Both WT and SOD1 rats were tested for signs of paralysis following repetitive injury. No deficits were seen in any group after weeks 1 and 2 following initial injury (Figure 2A, red circle) indicating that this injury is relatively mild and does not cause acute paralysis. On the other hand, after sustaining a third injury, WT and SOD1 TBI animals began to show signs of forelimb paralysis and decreased BBB scores, relative to their respective sham counterparts. TBI animals of both genotypes exhibited a gradual decrease in BBB scores following their third injury through their fifth and last injury (Figure 2A, green arrows). Interestingly, following their final injury, partial recovery was observed in both WT and SOD1 TBI rats (Figure 2A, blue bracket). WT TBI rats maintained mild, yet significant long term paralysis resulting from 5 weeks of injury.

SOD1 TBI rats, following a brief recovery period, exhibited significantly early disease onset in terms of forelimb BBB score decline, relative to SOD1 sham rats (Figure 2B). Shortened survival observed in SOD1 TBI rats, relative to SOD1 sham rats, did not reach significance when TBI rats were grouped as a whole (Figure 2C, *p* = 0.07). However, we previously reported that with this injury model, although administered a similar injury each time, rats display varying levels of phenotypic symptom severity and those rats with more severe functional deficits after injury exhibited the most severe brain pathology [19,20]. We therefore next explored whether SOD1 rats exhibiting a more severe early injury phenotype showed any exacerbation in ALS disease phenotype relative to those with a milder injury phenotype.

### 3.3. Rotarod Performance Distinguishes Between Mild and Severe Motor Deficits Resulting from Repeat Mild TBI

**Figure 3:**
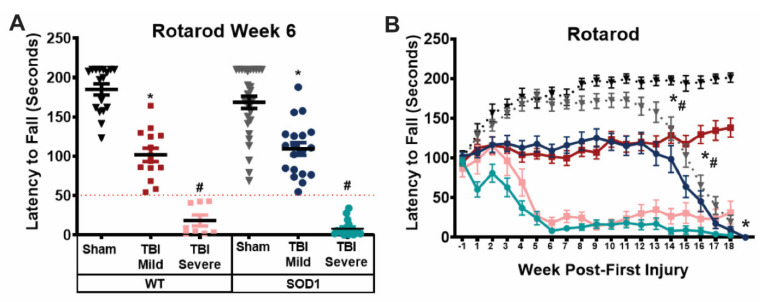
Rotarod performance distinguishes between mild and severe motor deficits resulting from repeat mild TBI. (**A**) TBI rats of each genotype were grouped based on symptom severity at week 6 post-first injury; those with a latency to fall shorter than 50 s on the Rotarod were designated “severe”, while those with a latency to fall longer than 50 s were designated “mild”. (* SOD1 Sham vs SOD1 TBI mild, * WT sham vs WT TBI mild, # SOD1 TBI Severe vs SOD1 Sham and SOD1 TBI mild, # WT TBI Severe vs WT Sham and WT TBI Mild, One-way ANOVA, *p* < *0*.05) (**B**) When grouped according to symptom severity, both WT and SOD1 animals with mild deficits performed significantly worse than sham animals on the rotarod, while those with severe deficits performed significantly worse than both sham and mild TBI animals of their respective genotype. SOD1 animals exhibited further decline in rotarod performance following disease onset. Deficits were sustained in WT TBI mild and severe animals beyond 21 weeks post-first injury. (* SOD1 Sham vs SOD1 TBI Mild vs SOD1 TBI Severe, * WT sham vs WT TBI Mild vs WT TBI Severe, #SOD1 Sham vs WT Sham, # SOD1 TBI Mild vs WT TBI Mild, Two-way ANOVA *p* < *0*.05).

Despite a uniform injury delivered weekly to each animal throughout the course of 5 weeks, variability in behavior deficits was observed such that animals could be divided distinctly based on their ability to maintain balance and motor function on the rotarod over time. Specifically, following the course of injuries, a group of TBI rats, including both WT and SOD1 animals, was observed to have only mild rotarod deficits, defined as maintaining their balance on the rotarod for more than 50 s. Another group of both WT and SOD1 TBI animals was observed to have more severe rotarod deficits, with difficulty performing the task and maintaining their balance on the rotarod for less than 50 s. Based on severity of rotarod deficits in the initial time period following their final injury, TBI rats were separated into “mild” and “severe” TBI groups for further analysis (Figure 3A). When stratified based on symptom severity at week 6 and assessed over time following this point, each sham/injury group, WT and SOD1 animals performed similarly on the rotarod until disease onset occurred in SOD1 groups. Sham rats exhibited a steady increase in latency to fall in the first 5 weeks of testing, likely due to a learned response. TBI animals in the mild groups of both genotypes failed to improve and performed significantly worse than sham controls. Rats in the severe TBI groups exhibited a decline in balance and motor function that decreased to a complete inability to remain on the rod (latency to fall less than 50 s) by week 6, following the full course of TBIs (Figure 3B).

### 3.4. SOD1 Rats with Initial Severe Symptoms Following Repeat TBI Exhibit Earlier Onset and Shortened Survival

**Figure 4:**
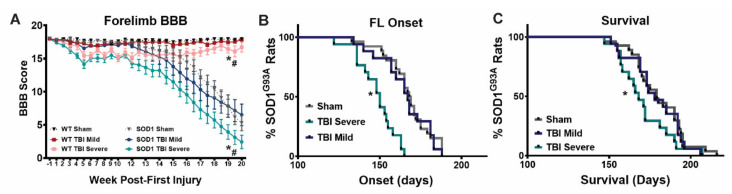
SOD1 rats with initial severe symptoms following repeat TBI exhibit earlier onset and shortened survival. (**A**) Forelimb BBB scoring revealed that WT TBI rats with initial severe symptoms maintain a significant, mild level of paralysis throughout the study, relative to both WT sham and mild TBI. When grouped by injury severity, SOD1 rats in the severe group experienced (**A**,**B**) an earlier decline in forelimb motor function and (**C**) shortened survival time relative to SOD1 rats in the sham and mild TBI groups. TBI rats exhibiting only mild initial symptoms did not differ from sham SOD1 rats in terms of disease onset and survival. ((**A**) * WT sham vs WT TBI Severe, * SOD1 Sham vs SOD1 TBI Severe, # SOD1 vs WT (all groups), Two-way ANOVA *p* < *0*.05. (**B**,**C**) Wilcoxon’s signed rank test * *p* < *0*.05).

As mentioned previously, rotarod scoring is a rather insensitive measurement of motor function that does not allow for evaluation of more fine-motor decline and paralysis. Within both mild and severe symptom severity groups on week 6 following final injury, wildtype and SOD1 TBI animals did not differ in rotarod scores. BBB scoring was therefore also used as a more sensitive assay to evaluate progressive forelimb decline in TBI animals that had been stratified based on symptom severity. No significant deficits were seen in any group after weeks 1 and 2 following initial injury, again, indicative that an experimentally mild TBI was being delivered. The SOD1 TBI severe group exhibited mild forelimb paralysis during weeks 3 to 5 of injury, and showed a partial recovery after the final injury, but then exhibited an earlier decline in motor function (Figure 4A) that resulted in earlier onset of forelimb paralysis (Figure 4B) and shortened survival times (Figure 4C), relative to SOD1 sham rats. On the other hand, those SOD1 rats that exhibited only mild symptoms in the acute post-injury time frame showed no difference in onset or survival time, relative to SOD1 sham controls, suggesting that more severe early behavioral deficits are predictive of a worsened long-term outcome. Given the BBB scoring data showing that SOD1 TBI rats in the severe group did not become completely paralyzed and in fact exhibited a degree of functional improvement following the last TBI prior to disease onset, this would indicate that severe TBI does not mask the effect on ALS. WT TBI animals in the severe group had significantly worsened forelimb BBB scores, relative to mild TBI and sham and maintained this mild paralysis throughout the study. This implies that severe injury can lead to long term mild paralysis in WT, however, these rats did not develop progressive disease-like paralysis as SOD1 rats (tested out to 54 weeks, data not shown), indicating that TBI is not causing ALS to develop in WT rats.

### 3.5. SOD1 Rats with Acute Severe Symptoms Following Repeat TBI Display an Early Loss of Corticospinal Motor Neurons

Given the clear correlation between severe injury symptoms in the acute time period after repetitive injury and exacerbated disease progression, we asked whether cortical pathology is altered and associated with severity of phenotype. We previously revealed that in SOD1 ALS rats, a significant reduction in large ( >300 µm) CTIP2+ cells is observed at disease endpoint relative to time points of 120 days and earlier [3] indicating a progressive degenerative upper motor neuron phenotype in this disease model. Here, we evaluated layer V corticospinal motor neurons (CSMN) in WT and SOD1 rats at two different time points after repetitive TBI. Brain tissue was collected and evaluated at study endpoint (P235 in WT and disease endpoint in SOD1 rats) as well as an “acute” time point, 48–72 h following the 5th and last TBI (~P90). Rats were again stratified into “mild” or “severe” groups based on injury symptoms in the acute phase after injury (24 h after the last injury for “acute” time point and week 6 for endpoint rats, as described above).

**Figure 5:**
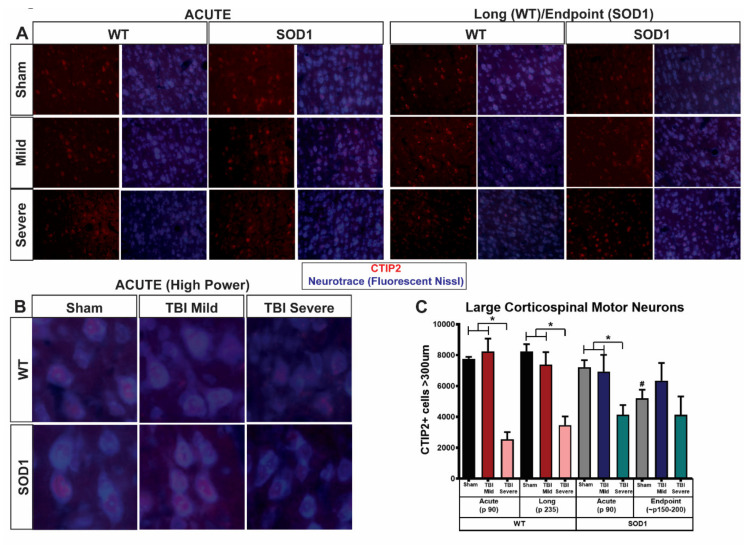
SOD1 rats with acute severe symptoms following repeat TBI display an early loss of corticospinal motor neurons. Immunostaining for CTIP2 and Neurotrace at (**A**) low and (**B**) high magnification showed a significant reduction and apparent disorganization of large CTIP2+ (COUP-TF-interacting protein 2) cells located in layer V of the motor cortex occurring in both WT and SOD1 TBI “severe” rats. Quantitative stereological analysis (**C**) revealed that this reduction occurred in the acute phase in only rats (both WT and SOD1) that exhibited early severe functional deficits and not those TBI rats that presented with mild phenotypic symptoms (* *p* < 0.05, error bars signify standard error of the mean, SEM). As expected, SOD1 sham endpoint animals exhibited a disease-related reduction in large CTIP2+ cells (>300 μm) relative to earlier timepoints (*p* < *0*.05) and this was not exacerbated with injury. Importantly, however, SOD1 rats experiencing early severe symptoms following repeat TBI showed an early reduction in large CTIP2+ cells at the acute time point not observed in SOD1 sham or mild rats at this time.

Following immunostaining for CTIP2 as a marker for CSMNs and evaluation of CTIP2+ cell size using fluorescent Nissl (Neurotrace, Figure 5A,B), we found that TBI rats showing severe symptoms are distinguished pathologically by those in the mild group by a reduction in large CTIP2+ cells in layer V of the motor cortex (Figure 5C). Quantitative stereological analysis of layer V CTIP2+ cells greater than 300 µm in size revealed that, as expected, degeneration of CSMN occurred at endpoint in sham SOD1 rats, relative to sham WT and sham SOD1 at the acute time point. SOD1 acute sham rats did not differ from WT acute sham rats indicating that this was a progressive degeneration, as reported previously [3]. TBI did not appear to exacerbate CSMN degeneration in SOD1 endpoint rats, as CSMN numbers remained similar at this time point between sham and mild as well as severe injury groups. On the other hand, WT rats at study endpoint in the severe group showed a reduction in the number of large CSMN relative to sham WT, and this difference was not observed in the mild TBI group. This reduction in large CSMN in the severe group was observed early, immediately following the 5th and final injury, as the acute TBI group with an early severe injury phenotype exhibited a significant reduction of large CSMN relative to both sham and mild TBI. Interestingly, this occurred similarly in both WT and SOD1 rats. SOD1 rats in the mild injury group, which did not show signs of exacerbated disease progression (Figure 4), did not exhibit changes in CSMN at the acute time point. On the other hand, SOD1 rats in the severe injury group with exacerbated disease progression exhibited early reduction of large CSMN linking poor corticospinal motor neuron health to earlier disease onset and earlier death in SOD1 rats.

There was no significant difference in the quantity of large CTIP2+ cells between WT and SOD1 animals within TBI groups. Importantly, there was no significant difference in the total number of CTIP2+ cells between groups, and TUNEL (terminal deoxynucleotidyl transferase dUTP nick end labeling) staining was absent in all groups (data not shown), suggesting neuronal atrophy, not death.

## 4. Discussion

In two independent studies, our laboratory established that beneficial effects are observed by directly therapeutically targeting the brain and the corticospinal motor neuron population in ALS, thus supporting the importance of upper motor neurons in initiating the cascade of events leading to motor circuitry breakdown in this devastating disease [3,21]. Here, in an alternative approach, we sought to determine whether an environmental stressor that directly alters brain health, such as TBI, exacerbates ALS in a genetically predisposed population. While we previously determined that a one-time, severe TBI does not affect disease progression in a rat model of ALS [22], we investigated the effects of recurrent TBI by developing a novel model of mild, repeat TBI whereby a closed-skull, bilateral CCI injury in rats delivered once weekly for 5 weeks, results in sustained motor deficits for at least 25 weeks [19,20]. Our model is unique in that these long-term deficits and the associated brain pathology have not been observed in other rodent TBI models that deliver either one-time or repeat unilateral insults.

Given the heterogeneous nature of head trauma, our bilateral closed-skull injury model results in a clinically relevant heterogeneous population whereby rats can be stratified based on injury-related symptom severity. Despite a uniform injury delivered weekly to each animal throughout the course of 5 weeks, variability in behavior deficits was observed such that animals could be divided distinctly based on their ability to maintain balance and motor function on the rotarod. While many TBI studies aim to achieve a clear, reproducible injury paradigm resulting in effects with low-variability, our model is advantageous and clinically relevant, as patients suffering from mild to moderate head injuries over time are unlikely to have a clearly defined history of diagnosed head trauma and are therefore treated based on symptom severity. Using this established model to more accurately represent injuries sustained by professional athletes and soldiers on a recurrent basis, we show that repetitive TBI is responsible for initiating premature onset of ALS in genetically predisposed ALS rats. While it is now widely accepted that populations prone to brain trauma have a higher risk for developing neurodegenerative disorders including ALS, this is the first time that an experimental link between repetitive head injury and earlier onset of ALS has been demonstrated, as well as a potential cause: degeneration of corticospinal motor neurons.

A single TBI in neonatal SOD1^G93A^ mice accelerated the later development of ALS [23], however single insults have been found to be insufficient to affect the pathogenesis of ALS in older animal models [22,24]. Clinical studies have shown a correlative relationship between brain trauma and ALS in populations prone to recurrent head injuries [25,26,27]. The bulk of repercussions of TBI are not a result of the primary injury to the brain, but are due to the secondary injury, which involves the cascade of events that lead to pathophysiology throughout the brain and central nervous system. This secondary injury is a response to primary injury that produces high levels of lactate, oxygen free radicals, interleukins, intracellular free Ca^2+^, and glutamate [28]. Recurrent trauma to the brain likely hinders full recovery from the secondary injury and leads to progressive damage over time. This supports the delayed appearance of clinical and pathological symptoms in CTE patients and is further validated in our model by the lack of tau upregulation, inflammation, and cortical atrophy in the acute phase following injury described previously [20]. Additionally, in this study, WT rats experiencing rTBI experienced a decline in in BBB scores, indicating weakened limbs, during weeks 3 to 5 of injuries, but showed recovery following the final injury. This is supported by clinical studies showing acute functional deficits followed by swift resolution of symptoms during the injury-free recovery period [29,30].

TBI and ALS share many pathological characteristics, including excitotoxicity, oxidative stress, inflammation, diffuse axonal injury, and apoptosis [31,32]. Cortical neuronal loss, corticospinal tract atrophy and ALS-like motor deficits were elicited in non-diseased young adult rats following fluid percussion injury [33]. Similarly, in the present studies, we found a reduction in large corticospinal motor neurons in WT rats experiencing rTBI, similar to that seen in untreated SOD1 rats at the initiation of disease onset. While various cell types are affected in ALS, signs of corticospinal motor neuron degeneration have been shown to be early events in ALS patients and rodent models [2,5,34]. Importantly in our studies, atrophy of corticospinal motor neurons was not enough to induce a more severe neurodegenerative motor phenotype in WT rodents, which were followed out to over 52 weeks with persistent motor deficits but no signs of progressive phenotypic decline (data not shown).

While the current studies lack a thorough investigation of the deeper mechanisms that clearly lead from repetitive trauma and atrophy of corticospinal motor neurons to worsened disease outcome, we set the stage for further exploration into how environmental stressors acting on the brain can influence disease onset and progression. Repeated trauma (but not a one-time injury) also exacerbated neurodegenerative phenotypes such as mortality and locomotor dysfunction in a Drosophila model of ALS [35]. Repetitive trauma in this model induced the formation of stress granules positive for ubiquitin and markers of other proteins implicated in ALS pathology. Involvement of stress granule formation has been linked to ALS [36] and modulation of stress granule formation has been shown to ameliorate disease pathology in ALS animal models [37]. Multiple other studies link stress granule assembly and RNA-binding proteins with prion-like domains to neurodegenerative disease [38,39,40]. TBI might alter protein clearance pathways whereby protein aggregation and the potential of prion-like spread are then compounded in a toxic ALS environment.

Impaired axonal transport of essential components such as mitochondria, RNA, and proteins has been implicated in ALS pathology [41], and has also been found to be induced by mild TBI [42,43]. As early as 3 days post-central fluid percussion injury, injured mice were found to have a reduction in the somatic area of Layer V pyramidal neurons positively expressing phospho-c-Jun, a marker for axotomized neurons [42]. Repetitive TBI-induced axonal injury could have an exacerbating effect on the already damaging impaired axonal transport seen in ALS and may contribute to the corticospinal motor neuron atrophy observed.

Energy metabolism is clearly disturbed following TBI [44]. Independent of TBI, it is also compromised in ALS patients and disruptions are known to contribute to ALS pathology [45,46,47]. Altered glucose metabolism leads to severe depletion of ATP in the central nervous system in ALS [48,49] and worsened disease outcomes have been linked to hypermetabolism [50]. Significant depletions in cortical ATP content in presymptomatic SOD1^G93A^ mice suggest that bioenergetic defects are involved in the initial stages of mSOD1-induced toxicity and imply that dysfunction within cerebral motor pathways precedes the selective dysfunction and degeneration of spinal cord motor neurons [51].

In the context of translational importance, in addition to silencing misfolded protein in the motor cortex using targeted AAV9-shRNA-SOD1 delivery, delivering healthy astrocytes producing GDNF to the motor cortex of ALS rats resulted in significant functional improvements [3,21]. Importantly, both strategies improved upper motor neuron health and had consequent downstream effects on spinal motor neurons suggestive of the potential to counteract the early development of brain-linked diseases and disorders caused by head trauma. Interventions to keep upper motor neurons healthy after trauma and in disease should continue to be investigated.

## 5. Conclusions

Given the existing commonalities between TBI sequela and known characteristic disruptions that occur in ALS disease pathology, it seems obvious that introducing premature cortical disruptions by means of TBI in those who are susceptible to ALS, would lead to an expedited disease time course. Our findings have important implications suggesting that, for genetically susceptible individuals, exposure to austere environments or participation in sports with a high incidence for repeat concussions should be minimized.

These studies are critical in highlighting the utility in combining our powerful model of repetitive TBI with a genetic model of neurodegenerative disease and the usefulness of this approach to explore repetitive TBI as a risk factor not only for ALS, but for other related diseases.

## Data Availability

Data is contained within the article or supplementary material.
